# Hemorrhagic Fever with Renal Syndrome Presenting with Hemophagocytic Lymphohistiocytosis

**DOI:** 10.3201/eid0802.010299

**Published:** 2002-02

**Authors:** Je-Jung Lee, Ik-Joo Chung, Dong-Hyeon Shin, Sang-Hee Cho, Duck Cho, Dong-Wook Ryang, Ali S. Khan, Hyeoung-Joon Kim

**Affiliations:** * Chonnam National University Medical School, Gwangju, South Korea; † Centers for Disease Control and Prevention, Atlanta, Georgia, USA

**Keywords:** Hantaan virus, Hemophagocytosis, Hemorrhagic fever with renal syndrome

## Abstract

Hemophagocytic lymphohistiocytosis--which is associated with a variety of infections, malignant neoplasms, autoimmune diseases, and immunodeficiencies--is an uncommon syndrome with a rapidly fatal outcome. We describe the first case of hemorrhagic fever with renal syndrome due to *Hantaan virus* presenting with reactive hemophagocytosis.

Hemophagocytic lymphohistiocytosis (HLH) is an uncommon syndrome characterized by a reactive, systemic proliferation of benign histiocytes throughout the reticuloendothelial system (*1*). It is associated with a variety of infections, malignant neoplasms, drugs, autoimmune diseases, and various immunodeficiencies. Infection-induced HLH is often associated with systemic viral infections, particularly Epstein-Barr virus, and occasionally with bacterial, fungal, or parasitic infections. For most patients with HLH, the outcome is rapid and fatal unless the diagnosis is made early and followed by prompt therapeutic intervention (*1*,*2*).

Hemorrhagic fever with renal syndrome (HFRS), which is caused by *Hantaan*, *Puumala, Seoul,* and *Dobrava-Belgrade viruses* (HTNV, PUUV, SEOV, and DOBV, respectively) is acquired primarily through aerosols of infectious rodent urine (*3*). Recently, Bart et al. (*4*) reported a case of hemophagocytic syndrome associated with PUUV, the most common cause of HFRS in Europe. Our report describes an unusual case of HFRS caused by HTNV presenting with secondary hemophagocytosis.

## Case Report

A 57-year-old woman was admitted to Chonnam National University Hospital, South Korea, with fatigue, generalized myalgia, and nausea of 2 weeks’ duration. Three weeks before admission, she had worked in a field in a rural area. Her vital signs were blood pressure 140/90 mm Hg, heart rate 80/min, temperature 36.6°C, and respiratory rate 20/min. On examination, she appeared acutely ill and had conjunctival suffusion, petechiae in the throat, an erythematous rash on the chest, tender hepatosplenomegaly, and mild tenderness in both flanks. There were no palpable lymph nodes.

Her leukocyte count was 3,200/μL, hemoglobin 9.9 g/dL, platelet count 25,000/μL, and reticulocyte count 0.3%. Blood chemistry revealed total serum protein 6.3 g/dL, albumin 3.0 g/dL, alkaline phosphatase 174 U/L, aspartate aminotransferase 369 U/L, alanine aminotransferase 175 U/L, total bilirubin 0.6 mg/dL (direct, 0.3 mg/dL), blood urea nitrogen 8.6 mg/dL, creatinine 0.5 mg/dL, lactic dehydrogenase 2,066 U/L, total cholesterol 100 mg/dL, HDL-cholesterol 22 mg/dL, triglyceride 285 mg/dL, and ferritin 20,000 μg/L. The coagulation profile included a prothrombin time of 13.6 seconds (control 12.5 seconds), a partial thromboplastin time of 45.2 seconds (control from 28 to 40 seconds), and a fibrinogen assay of 120 mg/dL. Serologic tests for viral infections--including antibodies against EBV, cytomegalovirus, herpes, *Hepatitis A, B,* and *C viruses*, and HIV--were negative. Serologic tests for Leptospira and *Rickettsia tsutsugamushi* and connective tissue diseases were also negative. HTNV titers using a particle agglutination kit (HANTADIA, Greencross, Korea) were 1:160 (normal <1:80). Cultures of blood, urine, and sputum were sterile. A computed tomographic scan of the abdomen showed moderate hepatosplenomegaly without lymphadenopathy. Bone marrow aspirate revealed proliferation of histiocytes with prominent hemophagocytosis (Figure). On day 8 of hospitalization, the second serologic titer for HTNV was elevated at 1:5,120. Fortunately, the patient recovered completely with only supportive care, including aggressive replacement of blood components, over 14 days (Table).

**Figure:**
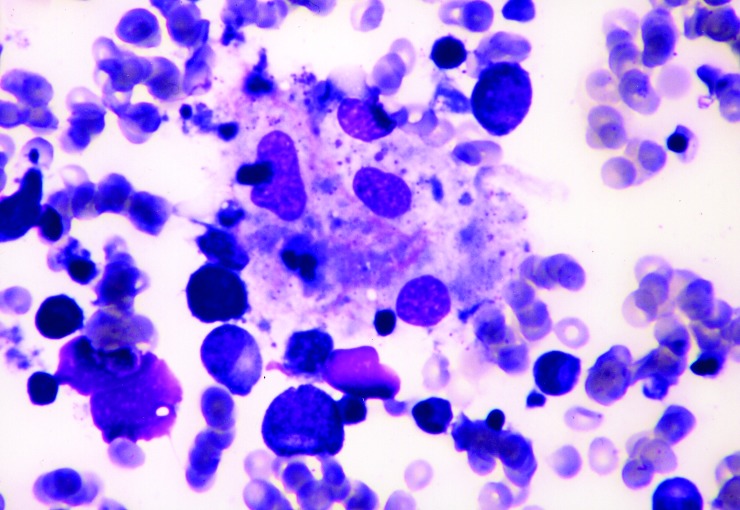
Bone marrow aspirate showing phagocytosis of neutrophil, nucleated erythrocyte, and platelets by benign histiocytes (Wright stain, x400).

**Table:** Serial laboratory findings in patient with hemorrhagic fever with renal syndrome from hospitalization until recovery, Korea

Laboratory test	On admission	Day 5	Day 8	Day 14
Leukocyte count (/μL)	3,200	3,300	4,100	4,400
Hemoglobin (g/dL)	9.9	10.2	11.4	11.2
Platelet count (/μL)	25,000	31,000	76,000	147,000
AST (U/L)	369	682	108	21
ALT (U/L) BUN (mg/dL) Cr (mg/dL)	175 8.6 0.5	433 8.8 0.8	205 8.6 0.7	21 13.6 0.9
Lactic dehydrogenase (U/L)	2,066	3,206	1,645	472
Ferritin (μg/L)	20,000	-	860	-
Hantaan virus titer	1:160	-	1:5,120	-

AST = aspartate aminotransferase, ALT = alanine aminotransferase; BUN = blood urea nitrogen.

## Conclusions

Patients with infection-associated HLH usually have persistent unexplained fever, cytopenia, lymphadenopathy, and, frequently, hepatosplenomegaly and coagulopathy, causing diagnostic difficulties with malignant histiocytosis or T-cell lymphoma (*2*). The possible immunopathologic mechanism of HLH might be excessive production of Th1 cytokines, such as gamma-interferon, tumor necrosis factor-alpha, interleukin-1, or interleukin-6, from activated lymphocytes or monocytes (*1*,*2*). Patients with Epstein-Barr virus-associated HLH (which if not treated is usually fatal because of hemorrhage, infection, or multiorgan failure) should initially be treated with a combination of corticosteroids and chemotherapy (*1*,*2*). As with our case, a patient with reactive HLH associated with organisms other than Epstein-Barr virus requires supportive care and treatment of the underlying disease (*1*,*2*).

We believe that this is the first case of HFRS caused by HTNV presenting with HLH. Thus, HFRS caused by HTNV or PUUV should also be considered as one of the underlying infectious diseases resulting in hemophagocytosis, requiring early diagnosis followed by prompt therapeutic intervention.

## References

[1] Imashuku S. Advances in the management of hemophagocytic lymphohistiocytosis. Int J Hematol 2000;72:1-11. [10979202[10979202

[2] Fisman DN. Hemophagocytic syndromes and infection. Emerg Infect Dis. 2000;6:601–8. 11076718 10.3201/eid0606.000608 PMC2640913

[3] McCaughey C, Hart CA. Hantaviruses. J Med Microbiol 2000;49:587-99. ]10882083] 10.1099/0022-1317-49-7-587 10882083

[4] Bart V, Schuhmacher H, Bourgoin C, Latger V, Buisine J, May T, Hemophagocytic syndrome and hemorrhagic fever with renal syndrome. Presse Med. 1998;27:1577. 9819588

